# The Neck-Persistency-Net: a three-dimensional, convolution, deep neural network aids in distinguishing vital from non-vital persistent cervical lymph nodes in advanced head and neck squamous cell carcinoma after primary concurrent radiochemotherapy

**DOI:** 10.1007/s00405-024-08842-3

**Published:** 2024-07-30

**Authors:** Matthias Santer, Philipp Zelger, Joachim Schmutzhard, Wolfgang Freysinger, Annette Runge, Timo Maria Gottfried, Andrea Tröger, Samuel Vorbach, Julian Mangesius, Gerlig Widmann, Simone Graf, Benedikt Gabriel Hofauer, Daniel Dejaco

**Affiliations:** 1 Department of Otorhinolaryngology-Head and Neck Surgery, Medical University of Innsbruck, 6020 Innsbruck, Austria; 2 Department for Hearing, Voice and Speech Disorders, Medical University of Innsbruck, 6020 Innsbruck, Austria; 3 Department of Radiation-Oncology, Medical University of Innsbruck, 6020 Innsbruck, Austria; 4 Department of Radiology, Medical University of Innsbruck, 6020 Innsbruck, Austria

**Keywords:** HNSCC, Radiomics, Artificial intelligence, Machine learning, Salvage surgery, PET/CT

## Abstract

**Purpose:**

To evaluate the diagnostic performance (DP) of the high-resolution contrast computed tomography (HR-contrast-CT) based Neck-Persistency-Net in distinguishing vital from non-vital persistent cervical lymph nodes (pcLNs) in patients with advanced head and neck squamous cell carcinoma (HNSCC) following primary concurrent chemoradiotherapy (CRT) with [18F]-fluorodeoxyglucose positron emission tomography and high-resolution contrast-enhanced computed tomography ([18F]FDG-PET-CT). Furthermore, the Neck-Persistency-Net’s potential to justify omitting post-CRT neck dissection (ND) without risking treatment delays or preventing unnecessary surgery was explored.

**Methods:**

All HNSCC patients undergoing primary CRT followed by post-CRT-ND for pcLNs recorded in the institutional HNSCC registry were analyzed. The Neck-Persistency-Net DP was explored for three scenarios: balanced performance (BalPerf), optimized sensitivity (OptSens), and optimized specificity (OptSpec). Histopathology of post-CRT-ND served as a reference.

**Results:**

Among 68 included patients, 11 were female and 32 had vital pcLNs. The Neck-Persistency-Net demonstrated good DP with an area under the curve of 0.82. For BalPerf, both sensitivity and specificity were 78%; for OptSens (90%), specificity was 62%; for OptSpec (95%), sensitivity was 54%. Limiting post-CRT-ND to negative results would have delayed treatment in 27%, 40%, and 7% for BalPerf, OptSens and OptSpec, respectively, versus 23% for [18F]FDG-PET-CT. Conversely, restricting post-CRT-ND to positive results would have prevented unnecessary post-CRT-ND in 78%, 60%, and 95% for BalPerf, OptSens and OptSpec, respectively, versus 55% for [18F]FDG-PET-CT.

**Conclusion:**

The DP of the Neck-Persistency-Net was comparable to [18F]-FDG-PET-CT. Depending on the chosen decision boundary, the potential to justify the omission of post-CRT-ND without risking treatment delays in false negative findings or reliably prevent unnecessary surgery in false positive findings outperforms the [18F]-FDG-PET-CT.

**Supplementary Information:**

The online version contains supplementary material available at 10.1007/s00405-024-08842-3.

## Introduction

Most patients with advanced head and neck squamous cell carcinoma (HNSCC) require multimodality treatment, including surgery followed by radiotherapy (RT) or primary concurrent chemoradiotherapy (CRT) [1, 2]. Restaging with high-resolution contrast-enhanced computed tomography (HR-contrast-CT) and/or [18F]-fluorodeoxyglucose positron emission tomography ([18F]FDG-PET) plus control biopsies from the primary tumor site, are routinely performed 8–10 weeks after treatment completion upon which the interdisciplinary tumor board (ITB) assesses remission [3]. Despite achieving complete remission at the primary tumor site, persistent cervical lymph nodes (LNs) are found in up to 40% after CRT, providing the rationale for a priori planned post-CRT neck dissections (NDs) [4–6]. However, post-CRT-ND is invasive, prone to complications, and cost-intensive [7, 8]. Thus, Mehanna and colleagues recommend clinical surveillance for [18F]FDG-PET-CT negative patients post-CRT, supported by evidence showing non-inferior survival rates compared to immediate post-CRT-NDs [8].

While [18F]FDG-PET-CT is more cost-effective than post-CRT-ND, its limited availability poses challenges, leading to the exploration of more accessible high-resolution contrast computed tomography (HR-contrast-CT) [3, 9]. The key challenge for both imaging modalities is to distinguish vital from non-vital persistent cervical LNs in post-CRT-ND with reasonable diagnostic performance [10–12]. Moreover, diagnostic performance must be tailored to individual patient comorbidities: for those eligible for post-CRT-ND, with selectively resectable persistent cervical LNs [13], optimizing sensitivity to avoid treatment delays is crucial, even at the risk of unnecessary surgery. Conversely, for patients with significant comorbidities, where only radical resection is possible, specificity should be emphasized to ensure that the benefits of surgery outweigh the risks of perioperative complications, by confirming the actual presence of histopathologically persistent cervical LNs.

Diagnostic accuracies of HR-contrast-CT and [18F]-FDG-PET-CT to distinguish vital from non-vital persistent cervical LNs after CRT, defining histopathology of post-CRT-NDs as reference, varied: HR-contrast-CTs were observed to be less effective compared to [18F]-FDG-PET-CT which sensitivities and specificities ranging from 16.7–89.0% and 89.0–97.1% respectively [14–19]. However, the largest retrospective study yet indicated that [18F]FDG-PET-CT did not improve the exclusion rate of persistent cervical LN compared to HR-contrast-CT alone. Thus, limiting post-CRT-ND to negative [18F]FDG-PET-CT results would have delayed treatment in 3 of 13 patients. Conversely, restricting post-CR ND to [18F]FDG-PET-CT positive finding would have prevented unnecessary surgeries in 11 of 20 patients [14].

In summary, neither HR-contrast-CT nor [18F]FDG-PET-CT alone are capable of sufficiently distinguishing vital from non-vital persistent cervical LNs after primary CRT. Currently, none of the imaging modalities can justify omitting post-CRT-ND without risking treatment delays, false negative findings, nor reliably prevent unnecessary surgeries in false positives findings. Consequently, alternative methods to distinguish vital from non-vital persistent cervical LNs in post-CRT HNSCC-patients need to be explored.

Radiomics has emerged as a promising method to enhance the diagnostic performance of HR-contrast-CTs [20] by extracting, processing, and analyzing quantitative data [21]. Key steps involve data acquisition, segmentation of the region of interest (ROI), feature extraction, and assignment to clinical outcomes (e.g. cervical LN persistency) through artificial intelligence (AI). AI includes machine learning, artificial neural networks (ANN), and deep learning (DL). DL, especially with a three-dimensional, convolutional, u-net architecture, uses convolutional kernels for efficient segmentation of complex images including HR-contrast-CTs. Despite the challenge of requiring large datasets, this can be addressed by transfer learning, which involves pre-training a neural network on available datasets and fine-tuning it for specific medical tasks. A systematic review of AI applications for LN classification in HNSCC-patients identified 13 retrospective studies, 9 of which used DL, mainly focusing on oropharyngeal and oral cancers [20]. DL has not been applied to distinguish vital from non-vital persistent cervical LNs in post-CRT HNSCC patients.

The present pilot study explored the diagnostic performance of a HR-contrast-CT three-dimensional, convolution, deep neural network—the Neck-Persistency-Net, a combination of an Autoencoder and fully connected neural network, specifically tailored to distinguish vital from non-vital persistent cervical LNs in post-CRT HNSCC-patients. Histopathologic finding of post-CRT-ND, performed in all study patients, was defined as a reference. The diagnostic performance of the Neck-Persistency-Net was compared to the best available published diagnostic performance for [18F]FDG-PET-CT for three scenarios: (a) balanced performance (BalPerf) for optimized sensitivity and specificity, (b) optimizing sensitivity (OptSens) in patients fit for post-CRT-ND and (c) optimizing specificity (OptSpec) in patients with high comorbidities. Besides exploring the diagnostic performance of the Neck-Persistency-Net, the key question to answer was how many HNSCC-patients with cervical LN persistency after primary-CRT received necessary surgery (i.e. vital persistent cervical LN were actually present in post-CRT-ND histopathology) and how many received unnecessary surgery (i.e. non-vital persistent cervical LN were found in post-CRT-ND histopathology) in order to assess the additional benefit of the Neck-Persistency-Net in this specific setting.

## Patients and methods

### Inclusion and exclusion criteria

Patients recorded in the institutional head and neck cancer registry at the Department of Otorhinolaryngology, Medical University of Innsbruck, Austria, between January 2008 and December 2023 were eligible. Inclusion criteria were incident, histologically proven, advanced HNSCC (UICC III or IV) including carcinoma of unknown primary; primary concurrent CRT; clinical response evaluation including neck and trunk HR-contrast-CT; ITB recommendation to perform post-CRT-ND due to certain or possible vital persistent cervical LN; primary tumor site disease-free or resectable and post-CRT-ND feasible with reasonable comorbidity.

Patients were excluded if they had cancer other than HNSCC; curative intent surgery was part of the first-line treatment; the ITB agreed on no evidence for vital persistent cervical LN after primary CRT; residual primary tumors or LN residual disease were considered not resectable; new-onset distant metastases were not accessible to curative intent treatment such as stereotactic body radiotherapy or surgery could not be performed due to the comorbidities.

### Staging and first-line treatment

All patients were staged according to the 8th Edition of the UICC Classification of Malignant Tumors [22]. Treatment recommendations were made by an ITB based on the results of a staging examination with an endoscopy under general anesthesia with a neck and trunk HR-contrast-CT in line with the recommendations of the National Comprehensive Cancer Network (NCCN) guidelines [3]. The primary CRT is based on Cisplatin (25mg/m^2^) for 4 consecutive days in 2 cycles or on a combination of mitomycin C 10mg/m^2^ and 5-flourouracil 1000mg/m^2^ 2 cycles. Radiotherapy was delivered using a linear accelerator. The planned target volume received a dose of 70 Gy in 35 fractions over 7 weeks, targeting both the primary tumor and pathologic lymph nodes. High-risk involved lymph node levels were treated with a dose of 60 Gy in 30 fractions, while low-risk elective levels received 54 Gy in 30 fractions. All patients were routinely restaged 8–10 weeks after completion of primary CRT. Treatment response evaluation included a neck and trunk HR-contrast-CT and restaging endoscopy under general anesthesia, with biopsies from the original tumor sites [15]. Treatment response evaluation was performed in accordance with the WHO recommendations [23, 24].

### CT-scans

HR-contrast-CTs were performed according to the head & neck imaging protocols of the Department of Radiology, Medical University of Innsbruck with GE-Medical Systems Light Speed VCT^®^ (GE Medical, Vienna, Austria), or Siemens SOMATOM^®^ Definition Flash or SOMATOM^®^ Drive (Siemens Healthineers, Erlangen, Germany). The scan area of the neck ranged from the frontal sinus to the upper mediastinum with a resolution of 512 × 512 pixels. Slices were calculated from raw data with 2 mm thickness, collimation of 24 × 1.2 mm, and 0.45 pitch. Additional coronal and sagittal slices were reconstructed. As a contrast medium, Jopamiro 370^®^ (Bracco Austria GmbH, Vienna, Austria) was administered intravenously. HR-contrast-CTs were exported in Digital Imaging and Communications in Medicine format using IMPAX EE^®^ (DICOM, Agfa HealthCare, Bonn, Germany) and the Picture Archiving and Communication System (PACS^®^, Cerner, Kansas City, USA). The HR-contrast-CTs were read by an experienced, board-certified head and neck radiologist and again reevaluated in the ITB. Criteria to classify LN as persistent cervical LNs after primary CRT were in line with Response Evaluation Criteria in Solid Tumors (RECIST).

### Segmentation

**Fig. 1:**
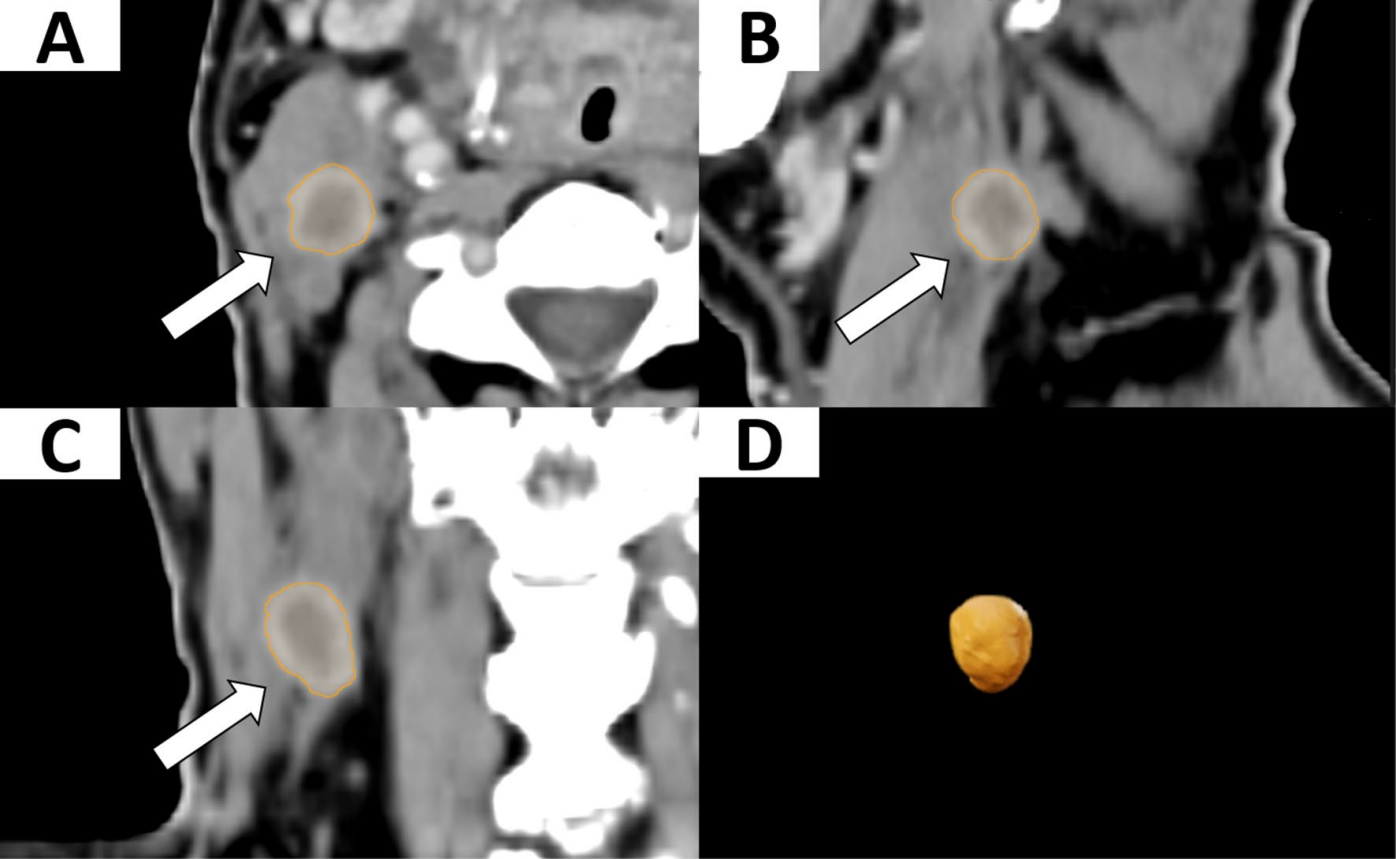
LN segmentation. Example of a persistent cervical LN with vital tumor cells of a 66-year-old, male HNSCC patient with a tumor of the Oropharynx staged cT1 cN2 cM0, p16 + . The segmentation is shown in axial plane (**A**); sagittal plane (**B**) and coronal plane (**C**), reformatted views and three-dimensional rendering are also shown (**D**) of the LN provided by the software. the white arrows indicate the segmented ROI in orange

For each of the included HNSCC-patients, the restaging HR-contrast-CT, was imported into the segmentation software Elements (Elements^®^, BrainLab, Munich, Germany). For each patient, the single certain or possible vital persistent cervical LN was manually segmented slice-by-slice in the axial, sagittal, and coronal plane as ROI. All segmented LNs were examined by, board certified head and neck radiologists with more than 15 years of clinical experience in head and neck HR-contrast-CT reporting (Fig. 1). The ROI, including quantitative data about shape, texture, and intensity (i.e. features) were exported further analysis by the Neck-Persistency-Net in DICOMDIR-format.

### Machine learning

#### Network architecture and training phase

The Neck-Persistency-Net, specifically tailored to the present study, applied a combination of unsupervised and supervised ML. Supervised ML involved training on labeled data, where the algorithm learned patterns and generated predictions based on input–output pairs. Unsupervised ML aimed at identifying patterns and structures in unlabeled data. Combining supervised and unsupervised methods enhanced model robustness by leveraging labeled data for targeted learning and unlabeled data for uncovering latent patterns, resulting in a more comprehensive and accurate Neck-Persistency-Net [25, 26].

The Neck-Persistency-Net applied in the present study relied on three-dimensional convolutions, which allowed in-depth access and analysis of radiomic data in all three spatial dimensions. Since the number of HNSCC-patients with certain or possible vital persistent cervical LN after primary CRT, even at a tertiary oncologic referral center, was limited, available data to train the Neck-Persistency-Net was sparse. This limitation was addressed with a convolutional neural auto-encoder architecture, which applied unsupervised ML to reconstruct input data (i.e. the high dimensional CT data) to the output data.

The term autoencoder describes the networks’ architecture, which resembles an “hourglass”. This “hourglass” consisted of (1) a wide top, representing high dimensional input data with numerous DL neurons (a base unit of a neural network, consisting of a non-linear function that received the input and calculated the weighted output, where the adaption of the weights during training, allowed the network to learn and make a prediction), referred to as “encoding phase”, (2) a narrow waist, representing a reduced representation (rrp) (similar to a principal component- or independent component analysis) of the high dimensional data with a limited number of DL-neurons, referred to as “latent space” and (3) a wide bottom, corresponding to the network output based on the reduced representation of the high dimensional input date with numerous neurons, referred to as “decoding phase” [27]. The architecture of the Neck-Persistency-Net designed for the present study is illustrated in Appendix A and B (App. A and App. B).

During the training phase, the Neck-Persistency-Net adjusted its weights in all layers to learn a reduced representation of the input data that aimed to retain the essential information of the input data in order to reconstruct the input data at the network's output (i.e. bottom of the hourglass or decoding phase). Since this auto-encoder-based training phase was performed by unsupervised ML, the high dimensional input data did not require the labels “vital” and “non-vital” persistent cervical LN. Thus, for the present study, random sections of the restaging HR-contrast-CT datasets of the neck were utilized. To generate additional layers of data variety, training data was augmented by rotations and mirroring of the HR-contrast-CT datasets.

After the unsupervised ML-based auto-encoding training phase, the bottom of the hourglass, or decoding part, was removed and all the weights of the encoder part, previously learned to generate the reduced representation of high dimensional data, were frozen. Hence, additional training of the Neck-Persistency-Net did not result in any additional alteration (i.e. weight refining) of the top and stem (i.e. decoder- and latent representation) of the algorithm.

Subsequently, supervised ML was introduced to the Neck-Persistency-Net by introducing additional classification layers (Appendix B). For the present study, two additional classification layers with 8 and 4 neurons using a ReLu activation function in both cases, respectively were introduced. These two classification layers with a total of 12 neurons, culminated in one single neuron that outputted the final classification result applying a sigmoid activation function, that generated numbers between “0” and “1”, where “0” corresponded to “non-vital” persistent cervical LN and “1” to “vital” persistent cervical LN after primary CRT.

**Fig. 2:**
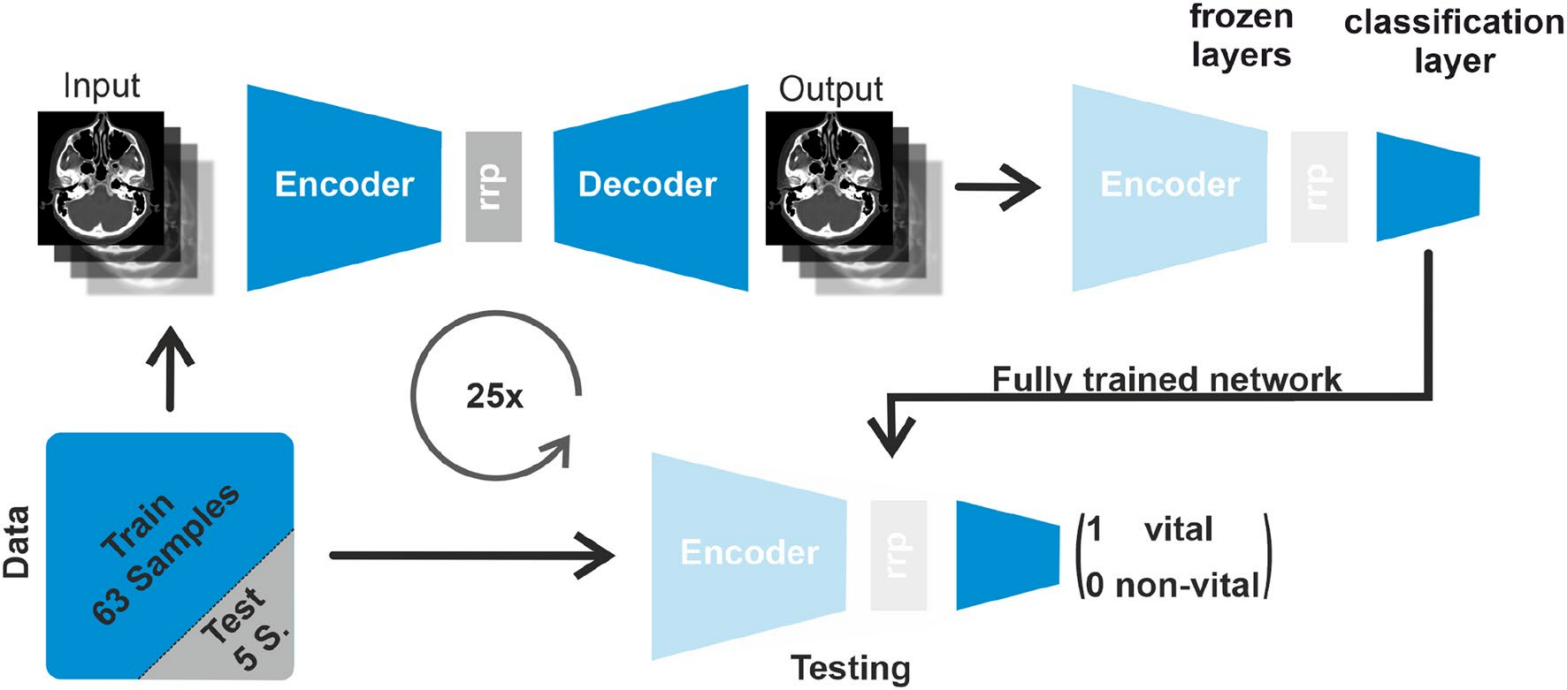
Schematic workflow of the training- and test phase. Schematic workflow of the training- and test phase of the DL algorithm designed to classify persistent cervical LNs after primary CRT as “vital” or “non-vital” based on histopathologic findings of post-CRT-ND. The data is separated into a training (blue) and a test set (gray). The training set is first used to train the autoencoder. After the trainings completion the weights of the autoncoder are frozen (i.e. they can not be changed by further training) and the decoder part is removed. In the next step classification layers are added to the trained encoder part. Those layers are trained in a supervised manner, to classify the input into vital and non-vital samples. After this second training step’s completion, the network is tested using the test data separated at the beginning. This process is repeated from scratch 25 times, by randomly choosing a different test/training data split (i.e. 25-fold-cross validation)

This introduction of supervised ML resembled a supplementary attachment to the Neck-Persistency-Net with significantly fewer parameters. Reducing the number of trainable parameters allowed us to train the classification part with significantly smaller datasets (i.e. the dataset of 68 patients). Finally, the supplementary attachment to the Neck-Persistency-Net was further trained with supervised, labeled ML aiming at an optimal distinction between “vital” and “non-vital” persistent cervical LN after primary CRT. During this phase, 25-fold cross-validation was performed, where 5 HR-contrast-CTs of 5 post-CRT HNSCC patients were randomly excluded from each training cycle. For each cycle, these 5 excluded datasets were neither included in the auto-encoder training- nor the training of the classification layers but reserved for network evaluation during the training’s conclusion. Each training cycle required approximately 20 min on an Nvidia RTX 3090 GPU (Nvidia^®^, Santa Clara, USA). The classification of one single dataset required less than one second. Figure 2 illustrates the schematic workflow of the training phase (Fig. 2).

In the first step of the training phase of the DL algorithm data is randomly split into training and test sets (63 and 5 datasets respectively). The training set is used to train an autoencoder in the first step. The weights from the autoencoder are frozen after the training, and the decoder part is replaced by classification layers. Those layers are trained to classify between “vital” and “non-vital” persistent cervical LNs after primary CRT. The final network is evaluated by the test data in the last step. For additional detail please refer to the main text.

#### Classifier evaluation and calculation of diagnostic performance parameters

The classifier evaluation was conducted using the Receiver Operating Characteristic (ROC), the Area under the curve (AUC), the true positive rate (TPR), the true negative rate (TNR), the false positive rate (FPR), and the false negative rate (FNR). TPR and TNR correspond to sensitivity and specificity, respectively, which were additionally calculated to facilitate comparability with previously published data for external validation. Moreover, NPV and PVV were calculated, as they account for the prevalence of a disease [10, 11],

The output of the Neck-Persistency-Net designed for the present study ranges from 0 to 1, whereas 0 corresponds to a “non-vital” and 1 to “vital” persistent cervical LN after CRT. The classification of an individual dataset as “vital” or “non-vital” depends on the previously defined decision boundary, i.e. the numerical threshold above or below which a dataset is categorized into either of the two categories. The ROC facilitates the evaluation of the Neck-Persistency-Net by allowing adjustment of the decision boundary across the range from 0 to 1 and therefore allowing to evaluate the classifier's abilities without pinning it down to a fixed decision boundary. A decision boundary set at 0.1 would emphasize sensitivity, with Neck-Persistency-Net outputs of > 0.1 would be classified as “vital”, while a decision boundary of 0.9 would emphasize specificity, with maximum Neck-Persistency-Net outputs of > 0.9 required to result in a “vital” classification.

An AUC of 0.5 corresponding to an ROC curve that follows a diagonal line from the top right to the bottom left indicates no discrimination. An AUC between 0.7 and 0.8 is considered Fair, while a range of 0.8 and 0.9 is deemed good, and an AUC surpassing 0.9 is considered excellent [28, 29].

A perfect classifier would be represented by an ROC curve extending from the right upper corner to the left upper corner and then dropping to the left lower corner, yielding an AUC of 1. Furthermore, the analysis of FPR and FNR were analyzed depending on the defined decision boundary.

### Ethical considerations

The study was approved by the review board of the Medical University of Innsbruck, Austria (1129/2023). All procedures conducted in these studies involving human participants were in accordance with the ethical standards of the institutional review board and with the Helsinki Declaration (1964) and its later amendments or comparable ethical standards.

## Results

### Study population

Patients with newly diagnosed carcinoma of the head and neck that were treated at the Department of Otorhinolaryngology, Head and Neck Surgery, Medical University of Innsbruck between January 2008 and December 2023 were consecutively included. Of these 68 patients were complying with the inclusion criteria. The mean age (± SD) of all patients was 61 (± 10) years. Most patients were male (57/68; 83.8%). After histological analysis of the ND specimens, 32 (47.1%) had vital (i.e. true cervical LN persistency) and 36 (52.9%) had non-vital persistent cervical LNs (i.e. complete nodal response) in histopathological examination (Table 1).

**Table 1:** Clinical characteristics of 68 HNSCC-patients with advanced HNSCC undergoing primary CRT

	Count	Percent
Sex		
Male	57	83.8%
Female	11	16.2%
Age		
≤ 50	12	17.6%
51–60	19	27.9%
61–70	26	38.2%
≥ 71	11	16.2%
Tumor site		
Oral cavity	7	10.3%
Nasopharynx	1	1.5%
Oropharynx	33	48.5%
Hypopharynx	9	13.2%
Larynx	4	5.9%
Nose/	1	1.5%
CUP^1^	13	19.1%
p16 (only for Oropharynx/CUP^1^)		
Positive	20	43.5%
Negative	26	56.5%
cT		
0	13	19.1%
1	9	13.2%
2	13	19.1%
3	16	23.5%
4	17	25.0%
cN		
0	0	0%
1	23	33.9%
2	40	58.8%
3	5	7.4%
Histopathological persistent cervical LN^2^ after primary CRT^3^		
Yes	32	47.1%
No	36	52.9%

^1^Cancer of unknown primary site; ^2^Lymph Node; ^3^Concurrent Chemoradiotherapy

### Machine learning

#### General performance of the Neck-Persistency-Net

**Fig. 3:**
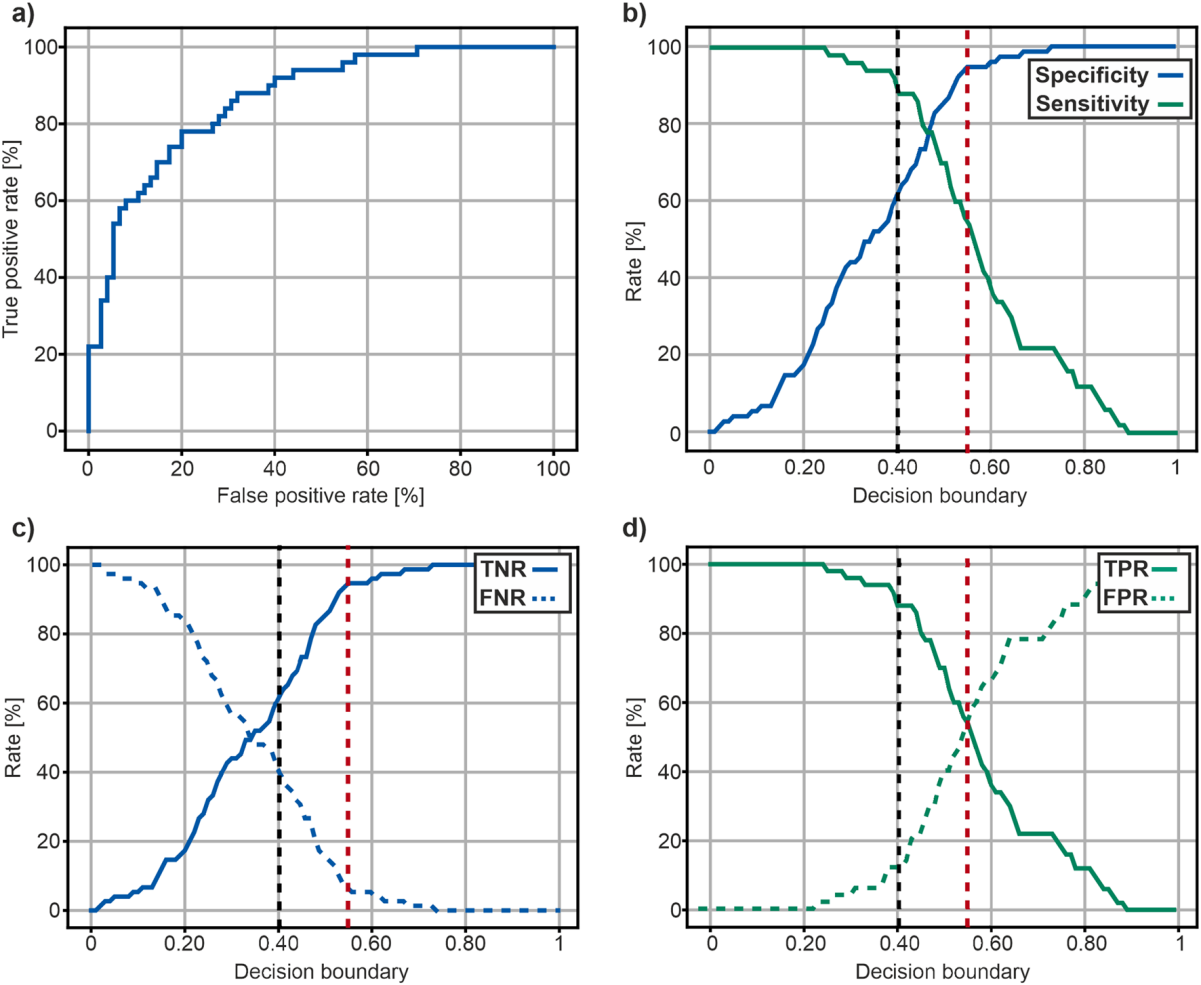
Diagnostic performance of the Neck-Persistency-Net. **a** depicts the ROC for the mean results yielded by the DL algorithm designed to classify persistent cervical LN after primary CRT in “vital” and “non” vital in 68 patients with advanced HNSCC. The y-axis represents the true positive rate (TPR) as percentage values, and the x-axis represents the false positive rate as percentage value. The blue curve represents the ROC curve. The AUC for the DL algorithm was 0.82, which corresponds to a good classifier (28). **b** illustrates the sensitivity and specificity yielded by the DL algorithm in dependence on the decision boundary. The y-axis represents the rate of correct classifications as percentage values, the x-axis represents the decision boundary ranging from 0 to 1. The dashed black line corresponds to a decision boundary at 0.41 with a sensitivity of 90% corresponding to a specificity of 62%. The dashed red line corresponds to a decision boundary at 0.55 and a specificity of 95% which leads to a sensitivity of 54%. A 95% sensitivity corresponds to 48% specificity, of the DL algorithm designed to classify persistent cervical LN after primary CRT in “vital” and “non-vital” in 68 patients with advanced HNSCC. **c** TPR and FPR and **d** TNR and FNR on the y-axis with respect to a moving decision boundary on the x-axis

The ROC curve yielded by the Neck-Persistency-Net resulted in an AUC of 0.82, which corresponds to a good classifier [29] (Fig. 3). Sensitivity and specificity pairs obtained by changing the decision boundary of the Neck-Persistency-Net are presented in Table 2 (Table 2) ranging from a specificity of 31% at 100% sensitivity to a specificity of 100% at 20% sensitivity.

**Table 2:** Decision boundaries

Sensitivity [%]	100	95	90	80	78	70	60	54	50	40	30	20	10	0
Specificity [%]	31	48	62	74	78	85	92	95	96	97	98	100	100	100

The table contains sensitivity and specificity pairs obtained by moving the decision boundary

#### Diagnostic performance of the Neck-Persistency-Net set to achieve an balance between sensitivity and specificity

In the first step, for comparison with available data about the diagnostic performance to distinguish vital from non-vital persistent cervical LN in post-CRT HNSCC-patients, the decision boundary of Neck-Persistency-Net was set to 0.47 in order to achieve an optimal balance between sensitivity and specificity. Thereby, a sensitivity of 78% and a specificity of 78% for the Neck-Persistency-Net, was observed with TPR of 78%, a FPR of 28%, a TNR of 78%, FNR of 27%, a NPV of 74% and a PPV of 71% (Fig. 3 and Table 2).

In terms of necessary or unnecessary surgery, this diagnostic performance translates to 25 of 32 patients who received necessary post-CRT-ND with histopathologically vital persistent cervical LN and 10 of 36 patients who received unnecessary post-CRT-ND with histopathologically non-vital persistent cervical LN.

#### Diagnostic performance of the Neck-Persistency-Net set to achieve an optimized sensitivity of 90%

In a second step, the decision boundary of the Neck-Persistency-Net was set to 0.41 in order to achieve an optimized sensitivity, comparable to the best previously reported sensitivity to distinguish vital from non-vital persistent cervical LNs in post-CRT HNSCC-patients aiming at patients eligible for post-CRT-ND, with selectively resectable persistent cervical LNs. If the decision boundary was set to 0.41 to achieve a sensitivity of 90%, the resulting specificity was observed at 62% (Fig. 3b). Thereby a TPR of 90%, an FPR of 13%, a TNR of 60%, an FNR of 40%, a NPV of 87%, and a PPV of 63%, were observed (Fig. 3 and Table 2).

In terms of necessary or unnecessary surgery, this diagnostic performance at a sensitivity of 90% translates to 29 of 32 patients who received necessary post-CRT-ND with histopathologically vital persistent cervical LN and 14 of 36 patients who received unnecessary post-CRT-ND with histopathologically non-vital persistent cervical LN.

#### Diagnostic performance of the Neck-Persistency-Net set to achieve an optimized specificity of 95%

In a third step, the decision boundary of the Neck-Persistency-Net was set to achieve an optimized specificity, comparable to the best previously reported specificity to distinguish vital from non-vital persistent cervical LNs in post-CRT HNSCC-patients [13] aiming at patients with significant comorbidities, where only radical resection is possible. If the decision boundary was set to 0.55 to achieve a specificity of 95%, the resulting sensitivity was observed at 54% (Fig. 3). Thereby a TPR of 54%, an FPR of 54%, a TNR of 95%, a FNR of 7%, a NPV of 94%, and a PPV of 47%, were observed based on this setting (Fig. 3 and Table 2).

In terms of necessary or unnecessary surgery, this diagnostic performance at a specificity of 95% translates to 20 of 32 patients who received necessary post-CRT-ND with histopathologically vital persistent cervical LN and 2 of 36 patients who received unnecessary post-CRT-ND with histopathologically non-vital persistent cervical LN.

## Discussion

Persistent cervical LNs after primary CRT in advanced HNSCC are observed in up to 40% of the patients [4]. Although a higher diagnostic performance to distinguish vital from non-vital persistent cervical LNs after CRT was observed for [18F]FDG-PET-CT and the more cost-effective compared to the post-CRT-ND, this imaging modality is not frequently available [3, 9, 30]. In addition, recent findings suggest that [18F]FDG-PET-CT did not improve the exclusion rate compared to HR-contrast-CT. Thus, neither HR-contrast-CT nor [18F]FDG-PET-CT can justify the omission of post-CRT-ND without risking treatment delays in false negative findings, nor reliably prevent unnecessary surgeries in false positive findings. Moreover, none of the currently available data considers individual patients comorbidities if deciding for or against post-CRT-ND: for patients fitted for post-CRT-ND, optimizing sensitivity to avoid treatment delays is crucial, even at the risk of unnecessary surgery. Conversely, for patients with significant comorbidities, specificity should be emphasized to ensure that the benefits of surgery outweigh the risks of perioperative complications, by confirming the actual presence of histopathologically persistent cervical LNs. Consequently, novel approaches to distinguish vital from non-vital persistent cervical LNs in post-CRT HNSCC-patients ought to be explored.

Radiomics is a promising novel method to enhance the diagnostic performance of HR-contrast-CTs [20] by applying AI. DL has not been applied yet for the specific task of distinguishing vital from non-vital persistent cervical LNs in post-CRT HNSCC-patients. The present pilot study explored the diagnostic performance of the Neck-Persistency-Net in the context of this specific task. The Neck-Persistency-Net was trained with a total of 68 patients with advanced HNSCC undergoing primary CRT and subsequent post-CRT-ND, whose histopathologic findings were defined as reference. Diagnostic accuracy was not only explored for a (a) balanced scenario with optimized sensitivity and specificity but also in the specific context of individual patients' comorbidities aiming at (b) optimizing sensitivity in patients fitted for post-CRT-ND, to avoid treatment delays, even at the risk of unnecessary surgery and (c) emphasizing specificity in patients with significant comorbidities, to ensure that the benefits of surgery outweigh the risks of perioperative complications.

The first key finding of the present study was that applying a HR-contrast-CT based, three-dimensional, convolution, deep neural network, referred to as “Neck-Persistency-Net” specifically tailored to distinguish vital from non-vital persistent cervical LNs in post-CRT HNSCC-patients, was feasible. Besides a “good” diagnostic performance with an AUC of 0.81 after only 25 training cycles, thus preventing overfitting, diagnostic accuracy for all three scenarios was comparable or outperformed to currently available diagnostic accuracies for [18F]FDG-PET-CT [14–19].

For the first scenario, the decision boundary of the Neck-Persistency-Net was set to 0.46, thus aiming at a balanced sensitivity and specificity of 78%, respectively. The observed sensitivity was approximately 11% lower than the maximum sensitivity previously reported for [18F]-FDG-PET-CT with 89.0%. The observed specificity was approximately 19% lower than the maximum specificity reported for [18F]-FDG-PET-CT with 97.1%. All in defining histopathology of post-CRT-NDs as reference [14]. In clinical terms, limiting post-CRT-ND to negative Neck-Persistency-Net results would have delayed treatment 9 of 32 patients (FNR 27%), a rate which is 4% higher than previously reported for [18F]FDG-PET-CT of 23% (3 out of 13 patients). Conversely, restricting post-CRT-ND to positive Neck-Persistency-Net results would have prevented unnecessary surgery in 28 of 36 patients (TNR 78%), a rate which is 23% higher than previously reported for [18F]-FDG-PET-CT of 55% (11 out of 20 patients). These observations suggest, that a balanced decision boundary of 0.46 of the Neck-Persistency-Net, aiming at an optimal balance between sensitivity and specificity, is comparable to [18F]-FDG-PET-CT in terms of treatment delay (27% vs. 23%) but remarkably outperforms [18F]-FDG-PET-CT in terms of preventing unnecessary surgery (78% vs. 55%) [14].

For the second scenario, the decision boundary of the Neck-Persistency-Net was set to 0.41, thus aiming at an optimized sensitivity of 90% for patients fit for post-CRT-ND to avoid treatment delays, even at the risk of unnecessary surgery. The observed specificity was 62%. Thereby, the sensitivity was approximately 1% higher than the maximum sensitivity previously reported at 89.0% and approximately 49% lower than the best-reported sensitivity for [18F]-FDG-PET-CT of 89.0% [14–19]. In clinical terms, limiting post-CRT-ND to negative Neck-Persistency-Net results would have delayed treatment in 12 of 32 patients (FNR 40%), a rate which is 13% higher than previously reported for [18F]FDG-PET of 23% (3 out of 13 patients). Conversely, restricting post-CRT-ND to positive Neck-Persistency-Net would have prevented unnecessary surgery in 32 of 36 patients (TNR 60%), a rate which is 35% higher than previously reported for [18F]FDG-PET of 55% (11 out of 20 patients). These observations suggest, that a decision boundary of 0.41 of the Neck-Persistency-Net, aiming at an optimized sensitivity of 90%, is inferior to [18F]-FDG-PET-CT in terms of treatment delay (40% vs. 23%) but remarkably outperforms [18F]-FDG-PET-CT in terms of preventing unnecessary surgery (60% vs. 55%) [14].

For the third scenario the the decision boundary of the Neck-Persistency-Net was set to 0.55, thus aiming at an optimized specificity of 95% for patients for patients with significant comorbidities to ensure that the benefits of surgery outweigh the risks of perioperative complications. The observed sensitivity was 54%. Thus, the specificity was approximately 2% lower than the maximum specificity previously reported at 97.0%, and the observed sensitivity was approximately 44% higher than the lowest reported specificity for [18F]-FDG-PET-CT [14–19]. In clinical terms, limiting post-CRT-ND to negative Neck-Persistency-Net results would have delayed treatment in 2 of 32 patients (FNR 7%), a rate which is 21% lower than previously reported for [18F]FDG-PET which was reported at 23% (3 out of 13 patients). Conversely, restricting post-CR ND to positive Neck-Persistency-Net would have prevented unnecessary surgery in 34 of 36 patients (TNR 95%), a rate which is 72% higher than previously reported for [18F]FDG-PET which was reported at 23% (11 out of 20 patients). These observations suggest, that a decision boundary of 0.55 of the Neck-Persistency-Net, aiming at an optimized specificity of 95%, is substantially inferior to [18F]-FDG-PET-CT in terms of treatment delay (7% vs. 23%) but remarkably outperforms [18F]-FDG-PET-CT in terms of preventing unnecessary surgery (95% vs. 55%) [14].

A key strength of any three-dimensional, convolution, deep neural network such as the Neck-Persistency-Net is its capacity to provide flexibility in choosing a diagnostic boundary. Clinicians are enabled to adjust the Nets decision boundary to meet the individual HNSCC-patients’ needs after primary CRT and possible persistent cervical LNs. In patients, fit for post-CRT-ND, the sensitivity can be optimized to avoid treatment delays, even at the risk of unnecessary surgery, while in patients with significant comorbidities, the specificity can be emphasized to ensure that the benefits of surgery outweigh the risks of perioperative complications. The currently available reference imaging modality of the [18F]-FDG-PET-CT does not provide comparable flexibility.

However, certain limitations of the present study have to be addressed: one of the key issues hampering the application of AI to enhance diagnostic performance for specific tasks such as distinguishing vital from non-vital persistent cervical LNs is its substantial demand for extensive datasets. Although in terms of AI, the total number of included HNSCC-patients of 68 is limited, this number represents the largest number of advanced HNSCC-patients undergoing primary CRT and subsequent post-CRT-ND currently available. The additional mitigation of this specific obstacle by transfer learning resulted in a “good” diagnostic performance of the Neck-Persistency-Net with a AUC of 0.81 [29]. This finding underlines a “good” diagnostic performance on one hand and a reliable transfer learning process on the other hand, which is prone to overfitting. Overfitting was observed between 25–50 training cycles, resulting in a Neck-Persistency-Net resistant to regularization, dropout, or pooling layers. Training was therefore concluded after 25 cycles to prevent the network from memorizing the training data due to the small dataset. Obviously, Neck-Persistency-Net’s full potential ought to be explored in a significantly larger sample of advanced HNSCC-patients undergoing primary CRT and subsequent post-CRT-ND. Restricting the input data to a single center, might introduce a theoretical bias into the training process.

Since the number of such specific patients, even at a tertiary oncologic referral center are limited in a next step a nation-wide multicenter study should be performed to refine the findings of the present pilot study. Thereby, the generalizability and robustness of the Neck-Persistency-Net might be improved.

Although findings of post-CRT imaging such as HR-contrast-CT are the decision marker for or against post-CRT-ND, other influential factors such as comorbidities are frequently considered by ITBs. A relevant proportion of advanced HNSCC-patients present with relevant comorbidities prior to primary CRT, which tend to deteriorate through the treatment thus rendering a significant limitation for post-CRT-ND [31, 32]. Although comorbidities were considered when changing the Neck-Persistency-Nets decision boundary, comorbidity surrogates such as the ASA score [33] were not provided as input data to the network. Besides a larger sample of HNSCC-patients to train and test the next work, a further development step should include the input of comorbidity data aiming at auto-adjusting the decision boundary to match the individual HNSCC-patients’ needs.

## Conclusion

Despite the various limitations, the Neck-Persistency-Net yielded promising results, underscoring the potential of neural networks to distinguish vital from non-vital persistent cervical LNs after primary CRT in patients with advanced HNSCC. The diagnostic performance of the network was comparable to the best available sensitivity and specificity data for the current reference imaging modality of [18F]-FDG-PET-CT. Depending on the chosen decision boundary the Neck-Persistency-Net’s potential to justify omission of post-CRT-ND without risking treatment delays in false negative findings or reliably prevent unnecessary surgery in false positive findings outperforms [18F]-FDG-PET-CT.

The present pilot study aims to pave the way for future research in this field. The positive outcomes achieved, even within the constraints of a limited dataset, highlight the significance of exploring and refining these approaches in larger, nationwide, multicenter studies.

## Supplementary Information

Below is the link to the electronic supplementary material.

Supplementary file1 (PNG 177 KB)

Supplementary file2 (PNG 133 KB)

## Data Availability

Additional data is available by contacting the corresponding author.

## Abbreviations

AUC: Area under the curve
AI: Artificial intelligence
ANN: Artificial neural networks
ASA: American Society of Anesthesiologists
BalPerf: Balanced performance
CRT: Chemoradiotherapy
DL: Deep learning
DP: Diagnostic performance
[18F]-FDG-PET-CT: [18F]-fluorodeoxyglucose positron emission tomography and high-resolution con-trast-enhanced computed tomography
FNR: False negative rate
FPR: False positive rate
GPU: Graphics processing unit
HNSCC: Head and neck squamous cell carcinoma
HR-contrast-CT: High resolution contrast computed tomography
ITB: Interdisciplinary tumor board
LN: Lymph node
ML: Machine learning
ND: Neck dissection
NPV: Negative predictive value
OptSens: Optimized sensitivity
OptSpec: Optimized specificity
pcLNs: Persistent cervical lymph nodes
PPV: Positive predictive value
RECIST: Response evaluation criteria in solid tumors
ROI: Region of interest
rrp: Reduced representation
SD: Standard deviation
TNR: True negative rate
TPR: True positive rate
UICC: Union for international cancer control
