# Supplementary figures and images for: The Neck-Persistency-Net: a three-dimensional, convolution, deep neural network aids in distinguishing vital from non-vital persistent cervical lymph nodes in advanced head and neck squamous cell carcinoma after primary concurrent radiochemotherapy
Source: Eur Arch Otorhinolaryngol. 2024 Jul 30;281(11):5971–82. doi: 10.1007/s00405-024-08842-3 (PMC11512899; doi:10.1007/s00405-024-08842-3)

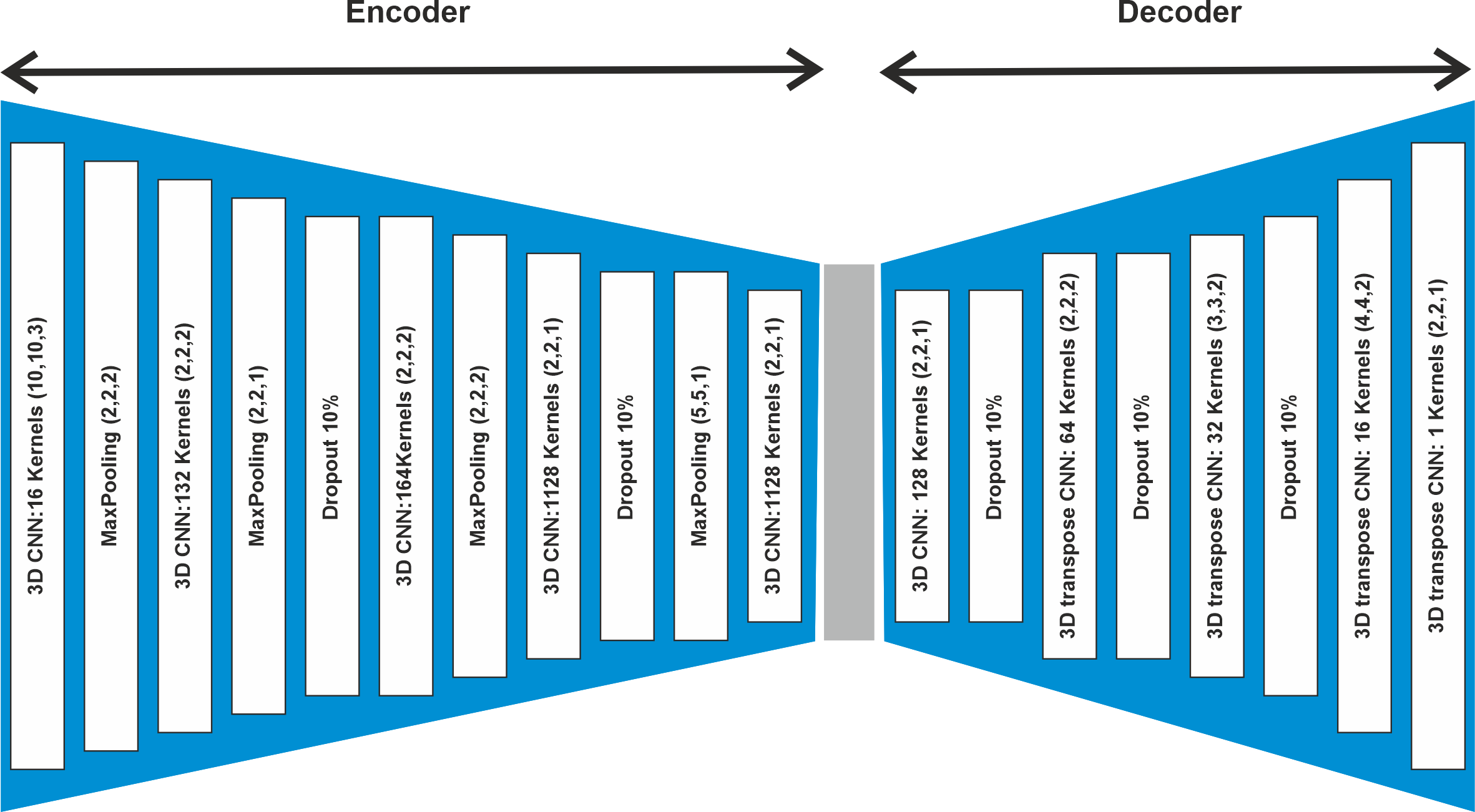

Supplement: Supplementary file 1 — Supplementary file1 (PNG 177 KB) [file 405_2024_8842_MOESM1_ESM.png]

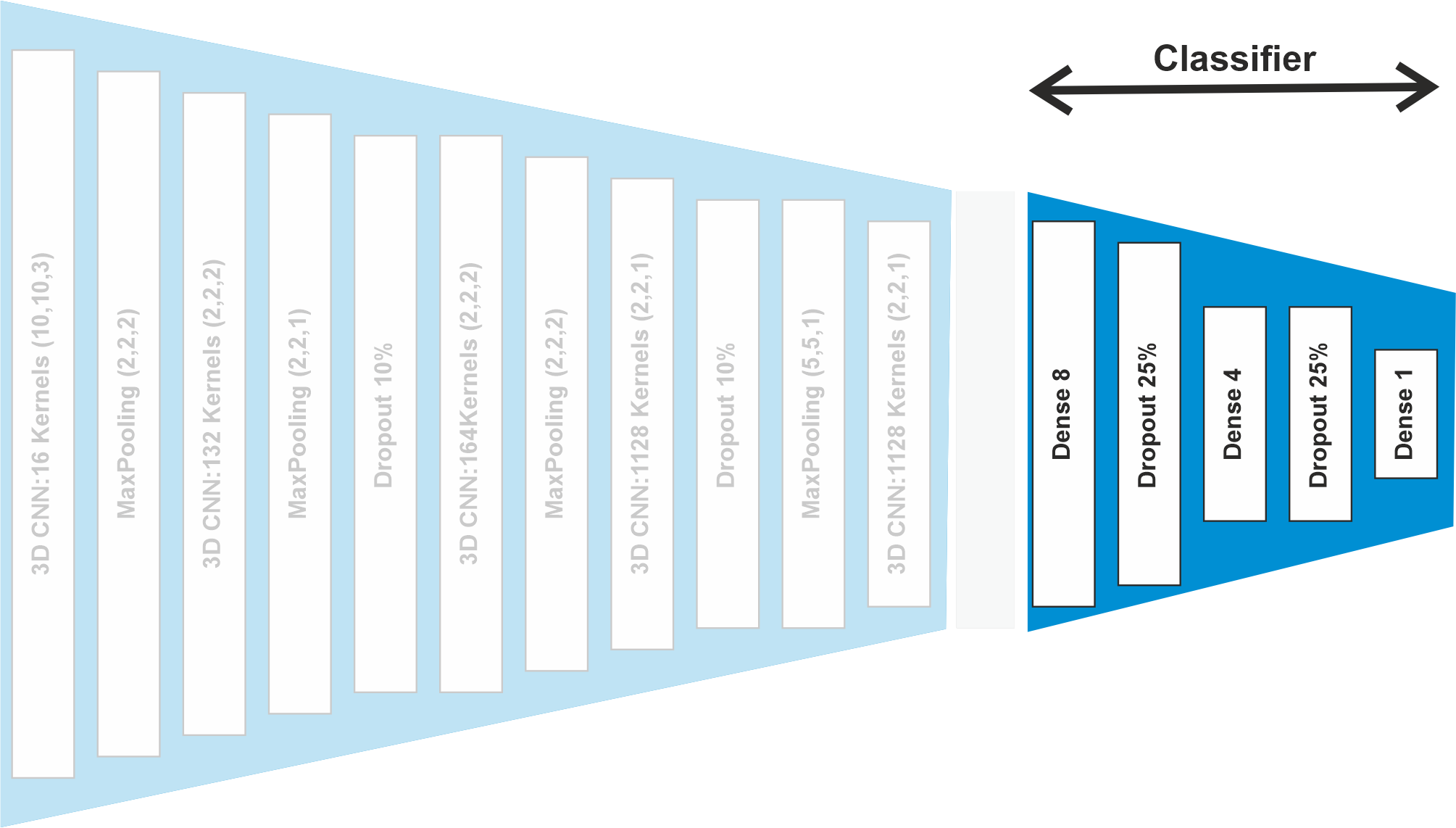

Supplement: Supplementary file 2 — Supplementary file2 (PNG 133 KB) [file 405_2024_8842_MOESM2_ESM.png]
